# Epidemiology of Lymphatic Filariasis Antigen and Microfilaria in Samoa, 2019: 7–9 Months Post Triple-Drug Mass Administration

**DOI:** 10.3390/tropicalmed9120311

**Published:** 2024-12-23

**Authors:** Helen J. Mayfield, Harriet Lawford, Benn Sartorius, Patricia M. Graves, Sarah Sheridan, Therese Kearns, Shannon M. Hedtke, Katherine Gass, Take Naseri, Robert Thomsen, Colleen L. Lau

**Affiliations:** 1 Centre for Clinical Research, The University of Queensland, Brisbane, QLD 4006, Australia; 2 College of Public Health, Medical and Veterinary Sciences, James Cook University, Cairns, QLD 4878, Australia; 3 School of Public Health and Community Medicine, University of New South Wales, Sydney, NSW 2033, Australia; 4 Menzies School of Health Research, Charles Darwin University, Casuarina, NT 0810, Australia; 5 Department of Environment and Genetics, La Trobe University, Bundoora, VIC 3086, Australia; 6 Neglected Tropical Diseases Support Centre, The Task Force for Global Heath, Decatur, GA 30030, USA; 7 Ministry of Health, Apia, Samoa

**Keywords:** lymphatic filariasis elimination, disease surveillance, Pacific region, *Wuchereria bancrofti*

## Abstract

The elimination of lymphatic filariasis (LF) as a public health problem remains an ongoing challenge in the Pacific region. This study reports on antigen (Ag) and microfilaria (Mf) prevalence in Samoa in 2019, 7–9 months after the completion of the first round of triple-drug mass drug administration (MDA). It evaluates the effectiveness of the intervention for reducing Ag prevalence to below a 2% threshold, and how this differs between 5–9-year-olds and ≥10-year-olds. We surveyed 30 randomly selected and five purposefully selected primary sampling units (PSUs) in Samoa in 2018 (1–3 months post-triple-drug MDA) and, again, in 2019. In each PSU, we conducted a community survey of 15–20 households and a convenience survey of 5–9-year-old children. A finger-prick blood sample was collected from all participants to test for Ag and Mf. Demographic details were also collected. There was no significant change in adjusted Ag prevalence in the 30 randomly selected PSUs between 2018 (3.9% [95% CI: 2.7–5.6%]) and 2019 (4.1% [95% CI 2.7–5.9%]). Significantly higher Ag prevalence was observed in participants aged ≥10 years (4.6%, 95% CIs 3.0–6.7%) compared to 5–9-year-olds (1.1%, 95% CIs 0.5–2.2%), supporting existing evidence that post-MDA surveillance should not be based on Ag prevalence among 6–7-year-olds. A single round of triple-drug MDA was insufficient to break LF transmission in Samoa 7–9 months post-MDA.

## 1. Introduction

Lymphatic filariasis (LF) is a vector-borne disease that can cause severe physical disfigurement including scrotal hydrocoeles and other irreversible lymphedema, contributing to mental health issues, social stigmatisation, and economic inequity [1]. The Global Program to Eliminate LF (GPELF) aims to eliminate LF as a public health problem by interrupting transmission in order to prevent new infections, manage morbidity, and improve the well-being and outcomes for those living with LF-related lymphedema. There have been notable successes with 17 endemic countries achieving elimination as a public health problem [2] and delivering over 8.6 billion treatments through GPELF between 2000 and 2020 [3].

The primary intervention strategy for LF elimination involves wide-scale treatment of the at-risk population through mass drug administration (MDA) programmes [4]. The World Health Organization (WHO) Neglected Tropical Diseases Roadmap 2030 [2] sets global milestones and targets to prevent, control, eliminate, and eradicate 20 neglected tropical diseases (NTDs), with this number growing to 21 in December 2023 [5]. To achieve the goal of eliminating LF as a public health problem, WHO recommends all LF endemic districts implement multiple rounds of high-coverage MDA, followed by post-treatment and, ultimately, post-validation surveillance. In addition, WHO requires all endemic areas to implement a minimum package of care for LF morbidity by 2030 [6].

Most commonly, MDA programs for LF elimination include multiple rounds of albendazole with either diethylcarbamazine (DEC) or ivermectin [1]. For countries where elimination has not been achieved with two-drug MDA, the WHO now recommends that ivermectin be distributed alongside DEC and albendazole (i.e., triple-drug MDA) for areas without endemic onchocerciasis [4]. Ivermectin has proven to be a safe and widely accepted treatment [7,8], and is effective in reducing the filarial load [9,10]. However, gaps remain in our knowledge regarding the effectiveness of a single round of triple-drug MDA in populations after an extended time period. Evidence from field trials conducted in Papua New Guinea have shown that triple-drug MDA was more effective than two-drug MDA in reducing microfilariae (Mf) prevalence [7,9], although a single round of triple-drug MDA was not sufficient to interrupt transmission [9]. Another study in Fiji observed no difference between triple-drug and two-drug MDA in a diurnally sub-periodic filarial transmission setting after 12 months [11].

The primary surveillance tool for determining whether MDA can be stopped is a school-based Transmission Assessment Survey (TAS) of children aged 6–7 years [1]. Thresholds for passing a TAS are parasite- and vector-specific. For *Wuchereria bancrofti* transmitted through an *Aedes* vector, passing a TAS requires the number of Ag-positive children identified in a survey to be below a given threshold, calculated so that the probability of an evaluation unit passing is at least 75% if the true Ag prevalence is 0.5%, with, at most, a 5% chance of falsely passing the TAS if the true Ag prevalence is ≥1% [1].

Antigenemia in younger children is more likely to be an indicator of recent infection, whilst antigenemia in older children and adults may be due to infections before MDA [1]. While sampling school children is considered convenient and cost-effective from a programmatic perspective, evidence suggests that Ag prevalence in younger children may not accurately reflect the Ag prevalence in the general population [12]. School-based TASs may therefore not be a suitable surveillance strategy for identifying ongoing LF transmission or determining elimination thresholds. Previous studies have shown that a TAS can fail to detect hotspot areas of residual transmission, especially where prevalence is higher in older age groups, and may not be as sensitive of an indicator of resurgence as older age groups [12,13].

In Samoa, all evaluation units failed a school-based TAS in 2017. This led to the roll-out of a nationwide triple-drug MDA in 2018, from 14–26 August, the first such intervention to be implemented by any country at a national level [14]. The decision to use a triple-drug MDA was based on Samoa’s long history of endemic LF, with multiple rounds of two-drug MDA under the Pacific Programme for Elimination of LF (PacELF) between 1998 and 2017 proving to be insufficient for LF elimination [13]. The 2018 triple-drug MDA achieved good coverage, with an estimated 80% of the population taking the medication [14].

There were two main aims of this study: first, to assess the impact of one round of triple-drug MDA against the previously reported baseline Ag prevalence [15]); second, to assess the spatial epidemiology of LF in Samoa post-triple-drug MDA. The specific objectives are listed as follows:

(i) Report on change in Ag prevalence from 2018 to 2019, 7–9 months post-triple-drug MDA;
(ii) Investigate Mf prevalence and the spatial epidemiology of LF in Samoa 7–9 months post-triple-drug MDA;
(iii) Compare Ag prevalence between age groups (age 5–9 years vs. age ≥10 years), sex (males vs. females), and randomly vs. purposively selected PSUs;
(iv) Compare geographic clustering of Ag positivity between 2018 and 2019, and between randomly and purposively selected PSUs.

This work expands on previously reported results [15,16] by including the complete dataset from 35 PSUs compared to the 28 reported in McPherson et al. (2022) [16], as well as the prevalence and epidemiology of the previously unreported 2019 Mf observations. The inclusion here of a detailed analysis of 2019 Ag prevalence by age, sex, and PSU selection (random vs. purposive), including analysis of the change in the clustering of Ag positivity, provides vital insights for informing targeted surveillance strategies and future MDA deployments.

## 2. Methods

### 2.1. Study Location

Samoa is a Pacific Island nation with a population of around 200,000 [17]. It has a tropical climate with an average rainfall of 3000–6000 mm/year [18]. There are two main islands, divided into four administration regions: three on the island of Upolu (Apia Urban Area [AUA], North West Upolu [NWU] and the Rest of Upolu [ROU]), and the fourth the island of Savai’i (SAV). The country is predominately rural with urban centres around Apia on Upolu and Salelolonga on Savai’i. The main vector associated with LF is *Aedes polynesiensis*, a day-biting mosquito that transmits the *W. bancrofti* parasite. In Samoa, the parasite is diurnally sub-periodic, meaning that Mf circulates in the peripheral blood at any time but in higher concentrations during the daytime [19].

### 2.2. Study Design

The first baseline survey was conducted from 26 September to 9 November 2018, with all participants enrolled within 3 months from the start of the first round of triple-drug MDA. The second survey took place in 2019 from 28 March to 17 May in the same 35 primary sampling units (PSUs). The first survey in 2018, which would ideally have been conducted prior to the 2018 MDA round, was delayed until 1 month after the MDA due to logistical reasons beyond the research team’s control. Since Ag is known to persist for at least months after treatment, the results of the 2018 survey were still expected to provide an accurate measure of pre-MDA Ag prevalence [15]. However, Mf is rapidly cleared after treatment, so Mf prevalence in our 2018 survey would likely to have been much lower than pre-MDA levels. The timing for the second survey in 2019 was based on recommendations from WHO [4] that surveillance should be conducted at least 6 months after the first effective round of triple-drug MDA. PSUs were sampled in approximately the same order in both years, resulting in approximately 6 months between the first and second surveys in each village.

Thirty PSUs were randomly selected and the remaining five were purposively selected by the Samoa Ministry of Health as suspected hotspots, based on the results of previous surveys. Participant recruitment and target sample sizes have been described in detail elsewhere [15]. Briefly, from each PSU, we aimed to recruit 57 participants aged ≥10 years and 57 participants aged 5–9 years. Sample sizes were calculated to detect 2% Ag prevalence in each age group, with a 5% chance of type 1 error, 75% power (when true prevalence is 1%), and a design effect of 2.0.

In 2018 and 2019, 15 households were selected per PSU, using a virtual walk method [15]. Although houses were randomly selected each year, by chance, a small number of houses were likely enrolled in both surveys. Each household was visited at least once between 3 pm and 8 pm, Monday–Saturday. If the selected building was not a house, or the residents declined to participate, it was replaced with the nearest household. If no one was at home, field teams made a second visit where possible. If the target sample size for participants aged ≥10 years was not reached after visiting every selected household at least once, houses where nobody was at home were replaced. If there were still insufficient participants after 15 households were surveyed, up to five additional households were randomly selected.

In each PSU, a convenience survey of 5–9-year-old children was conducted. Surveys took place within the PSU, generally at a community location such as a church, school, or the household of a community leader. The convenience surveys were arranged in collaboration with the village mayor and with the permission of the village Women’s Committee. If insufficient children were recruited after the first visit, either a second session was organised or the field teams randomly selected additional households within the village to recruit participants in this age group.

### 2.3. Data Collection

Surveys were conducted by community workers from the Samoa Red Cross Society in collaboration with the research team. Field teams collected the GPS coordinates of each household enrolled in the community surveys using smartphones. Demographic information was collected from each consenting participant using a standardised electronic questionnaire with standard data kit (SDK) software (https://www.datastandard.co, Accessed 1 March 2019). A clinical examination was conducted for scabies (including residents of all ages), the results of which have been reported elsewhere [20]. During the convenience surveys, field teams collected demographic data from a child’s parent or guardian using a short electronic questionnaire. For all participants aged ≥5 years, a finger-prick blood sample (up to 400 μL) was collected into a heparin microtainer.

### 2.4. Sample Processing

Blood samples were kept cool using ice packs until they were refrigerated upon the field teams’ return to the field laboratory. Samples were processed within 48 h of collection. Blood samples were brought back to room temperature before being tested for Ag, using Alere^®^ Filariasis Test Strips (FTS) (Scarborough, ME, USA), which were read at 10 min. Any blood samples that tested Ag-positive were used to prepare up to three thick blood slides, each with three 20 μL lines of blood, as per WHO guidelines [1]. Slides were dehaemoglobinised in water for 10–15 min and left to dry for 72 h. Two of the three slides were fixed with 100% methanol and stained with Giemsa, according to WHO-recommended methods [1], and read by independent readers (one slide per reader). A sample was considered Mf-positive if either reader identified Mf on any of the slides.

### 2.5. Data Analysis

Ag and Mf prevalence for 2018 and 2019 were estimated at national, regional, and PSU levels. Estimates were calculated using the Stata version 17 (Stata Corp, TX, USA) proportion command for each age category (5–9 and ≥10 years), and adjusted for selection probability at PSU and household levels and clustering at PSU level. Results were standardised by age group and gender against matching 2016 census distributions and adjusted for survey design. The presence/absence of Ag and Mf were mapped at the PSU level. Mf density (Mf/mL) for each Mf-positive participant was calculated as the average number of Mf per 60 µL of blood observed on each slide, converted to Mf/mL. Mean Mf density for the population was calculated as the geometric mean (due to skewed distributions) of the Mf/mL density for all Mf-positive participants in 2019.

To examine changes in Ag prevalence over time, at the national level, we fitted multivariable mixed effect logit models with random/group effects for each PSU at baseline (2018) and random/group effects for the temporal change at the PSU level. To examine changes over time by region, we modified the model to include fixed/population effects for each region in 2018 and fixed/population effects for the temporal change. From these models, we calculated odds ratios (ORs) and 95% confidence intervals (CIs) to determine significant changes in prevalence between years. The geographic clustering of Ag at the household, PSU, and regional levels were assessed using the intra-cluster correlation coefficient (ICC), estimated using the same multi-level mixed effects logistic regression model formulation, adjusting for age and sex and compared between years. Because the 2018 survey was conducted post-MDA, a comparison of Mf prevalence between 2018 and 2019 was not appropriate.

### 2.6. Role of the Funding Source

The study funder had no role in study design, data collection, data analysis, data interpretation, the writing of the report, or the decision to submit the results for publication. The corresponding author had full access to all of the data in this study, and had final responsibility for the decision to submit for publication.

## 3. Results

### 3.1. Demographics of Study Population

The demographic characteristics of the study population in 2018 [15] and 2019 are summarised in Table 1. In 2019, 4290 participants were recruited across household (*n* = 2629) and convenience surveys (*n* = 1661). The mean age was 20.3 years (range: 5–89); 52% of participants were female. A total of 3654 participants were recruited from the 30 randomly selected PSUs, and 636 from the five purposively selected PSUs. The mean number of households per PSU was similar between randomly selected (15.7) and purposively selected PSUs (15.0).

### 3.2. Adjusted Antigen Prevalence in 2019

**Figure 1:**
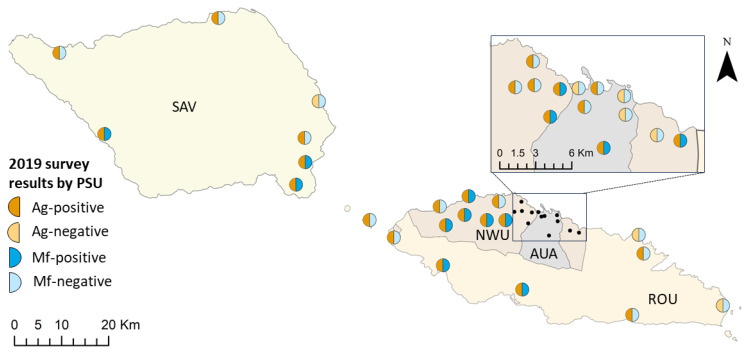
Observed Ag and Mf infection at the PSU level in Samoa in 2019. Regions shown are Apia urban area (AUA), North-west Upolu (NWU), Rest of Upolu (ROU), and Savai’i (SAV). Spatial data on country, island, region, and village boundaries in Samoa were obtained from the Pacific Data Hub (pacificdata.org, accessed on 8 July 2020) and DIVA-GIS (diva-gis.org, accessed on 12 August 2019) under an open access licence available at https://pacific-data.sprep.org/resource/public-data-license-agreement-0 (accessed on 12 August 2019).

Of the 4290 participants in 2019, a valid FTS result was available for 4256 (99.2%) participants, of which 139 (3.3%) were Ag-positive. Ag-positive PSUs (PSUs with at least one Ag-positive participant) were identified in all four regions (Figure 1). Adjusted Ag prevalence in the 30 randomly selected PSUs was 4.1% (95% CIs 2.7–5.9%), and there was a significantly higher Ag prevalence in purposively selected PSUs (14.9%, 95% CIs 13.7–16.0%; *p* < 0.001). Patterns of Ag-positivity in the random PSUs were similar to those observed in 2018; significantly higher Ag prevalence was seen in participants aged ≥10 years vs. 5–9 years (4.6%, 95% CIs 3.0–6.7% vs. 1.1%, 95% CIs 0.5–2.2%; *p* < 0.001), in males vs. females (6.7%, 95% CIs 4.4–9.8% vs. 1.3%, 95% CIs 0.5–2.7%; *p* < 0.001). For the 30 randomly selected PSUs, Ag prevalence was highest in SAV (10.2%) and lowest in AUA (1.5%). Full details of Ag prevalence, including by region, are provided in Supplementary Tables S1 and S2.

### 3.3. Adjusted Microfilaria Prevalence in 2019

In 2019, 32 (0.8%) participants tested Mf-positive. Mf-positive PSUs (PSUs with at least one Mf-positive participant) were identified in all four regions (Figure 1). Adjusted Mf prevalence in the 30 randomly selected PSUs was 0.8% (95% CIs 0.3–1.6%), with an average Mf density (geometric mean) among Mf-positive participants of 138.7 Mf/mL (min = 8.3, max = 2558.3, median = 170.8, IQR = 250.0). A significantly higher prevalence of Mf-positives was found in purposively selected vs. randomly selected PSUs (4.3%, 95% CIs 3.4–5.4% vs. 0.8%, 95% CIs 0.3–1.6%; *p* < 0.001), in participants aged ≥10 years vs. those aged 5–9 years (0.9%, 95% CIs 0.3–1.8% vs. 0.1% 95% CIs 0.0–0.8; *p* = 0.045), and in male vs. females (1.3%, 95% CIs 0.5–2.8% vs. 0.3%, 95% CIs 0.1–0.8; *p* = 0.007). Full details of Mf prevalence, including by region, are provided in Supplementary Tables S2 and S3.

### 3.4. Change in Ag from 2018 to 2019

**Figure 2:**
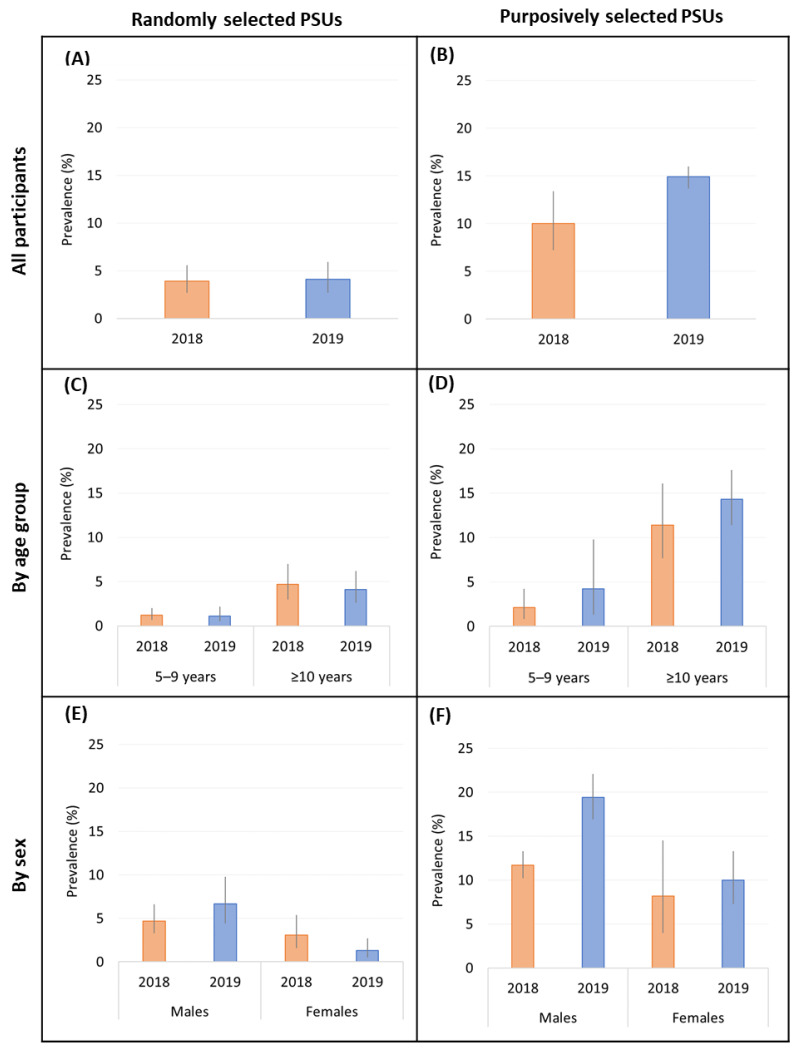
Adjusted antigen prevalence in randomly selected and purposively selected PSUs in 2018 [orange] and 2019 [blue] for all participants (**A**,**B**), stratified by age (**C**,**D**) and sex (**E**,**F**).

In the randomly selected PSUs, there was no change in the adjusted Ag prevalence from 2018 (3.9%) to 2019 (4.1%). By age group, Ag prevalence was unchanged in 5–9-year-olds from 2018 to 2019 (1.2% vs. 1.1%; *p* = 0.793) and in ≥10-year-olds (4.7% vs. 4.1%; *p* = 0.547) (Figure 2C). There was an insignificant increase in Ag prevalence among male participants between 2018 and 2019 (4.7 vs. 6.7%; *p* = 0.361) and an insignificant decrease among female participants (3.1% vs. 1.3%; *p* = 0.052). Confidence intervals are reported in Supplementary Table S1.

In purposively selected PSUs, the adjusted Ag prevalence did not change significantly from 2018 (10.0%) to 2019 (14.9%; Figure 2A,B). By age group, Ag prevalence was not significantly different among 5–9-year-olds (2.1% vs. 4.2%; *p* = 0.074) and ≥10-year-olds (11.4% vs. 14.3%; *p* = 0.200) between 2018 and 2019 (Figure 2C,D). Between 2018 and 2019, Ag prevalence for male participants (11.7% vs. 19.4%; *p* = 0.077) and female participants (8.2% vs. 10.0%; *p* = 0.234) did not change significantly (Figure 2E,F). Confidence intervals (95%) are reported in Supplementary Table S3.

### 3.5. Odds of Ag Positivity by Year

**Figure 3:**
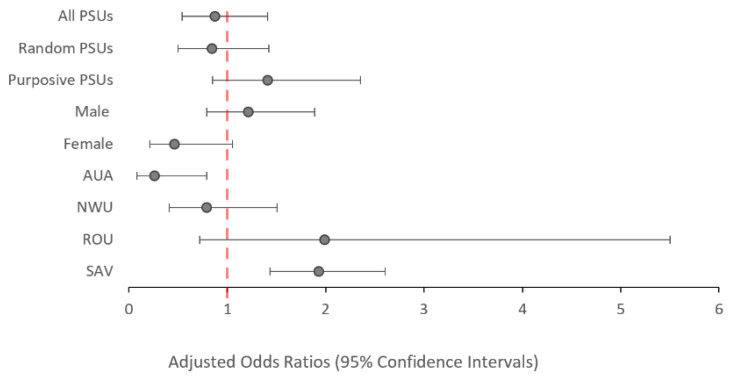
Adjusted odds ratios of testing positive to antigen in 2019 compared to 2018 (reference value) among all participants aged ≥5 years, stratified by PSU selection, sex, and region. Values above 1.0 indicate and increased odds of testing antigen-positive. Values below 1.0 indicate a reduced odds of testing antigen-positive.

There was no significant change in the odds of testing Ag-positive in 2019 compared to 2018 (aOR: 0.87; *p* = 0.573), irrespective of whether participants were from purposively selected PSUs (aOR: 1.41; *p* = 0.135) or randomly selected PSUs (aOR: 0.84; *p* = 0.498), as shown in Figure 3. At the regional level, participants from AUA had significantly lower odds of testing Ag-positive in 2019 vs. 2018 (aOR: 0.26; *p* = 0.026), while participants from SAV had significantly increased odds of testing Ag-positive in 2019 vs. 2018 (aOR: 1.93; *p* = 0.002). Confidence intervals (95%) are reported in Supplementary Tables S2 and S3.

**Figure 4:**
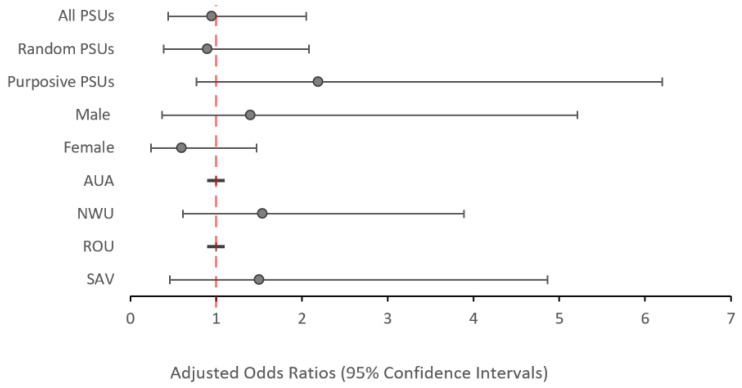
Adjusted odds ratios of testing positive to antigen in 2019 compared to 2018 (reference value) among all participants aged 5–9 years stratified by PSU selection, sex, and region. Values above 1.0 indicate an increased odds of testing antigen-positive. Values below 1.0 indicate a reduced odds of testing antigen-positive. There were no Ag-positive cases in AUA or ROU in 2019 for this age group.

**Figure 5:**
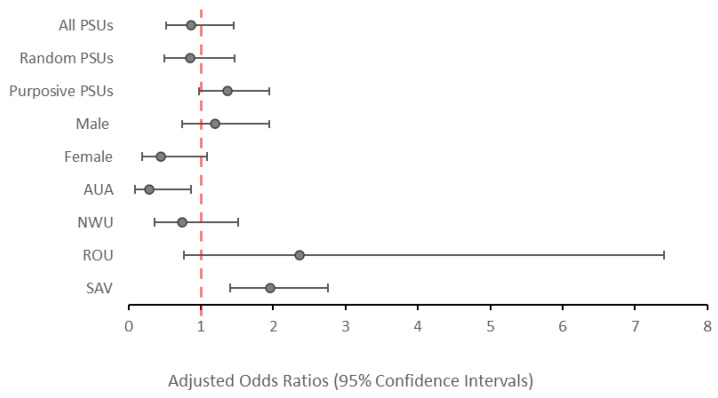
Adjusted odds ratios of testing positive to antigen in 2019 compared to 2018 (reference value) among all participants aged ≥10 years stratified by PSU selection, sex, and region. Values above 1.0 indicate an increased odds of testing antigen-positive. Values below 1.0 indicate a reduced odds of testing antigen-positive.

When all 35 PSUs were considered, there was no significant change in the odds of testing Ag-positive between 2018 and 2019 among participants aged 5–9 years (aOR: 0.95; *p* = 0.894) (Figure 4) or among participants aged ≥10 years (aOR: 0.86; *p* = 0.573). However, participants aged ≥10-years from AUA had significantly lower odds (aOR: 0.28; *p* = 0.033), while those from SAV had significantly higher odds (aOR: 1.96; *p* = 0.003) of testing Ag-positive in 2019 vs. 2018 (Figure 5).

### 3.6. Clustering of Ag Participants

As with the 2018 survey, the ICC of Ag-positive participants across the 35 PSUs in 2019 was highest at the household level (ICC 0.51), suggesting a high degree of clustering within households compared to the PSU (ICC 0.23) or regional level (ICC 0.02). There was no significant change in clustering between years at PSU, regional, or household levels. These results were consistent for both the 30 randomly and five purposively selected PSUs, as well as when calculated for the combined 35 PSUs (Supplementary Table S4).

## 4. Discussion

Ag prevalence in Samoa had not fallen to below 2% within 7–9 months after the first round of triple-drug MDA, which was distributed in 2018. Results show evidence of ongoing transmission, indicating that further intervention is needed to achieve elimination. This is in line with the current WHO recommendations that advocate multiple rounds of MDA [4]. Ag prevalence was lower in the younger vs. older age group (aged 5–9 vs. ≥10 years) and in the randomly selected vs. purposively selected PSUs.

Of the four regions, only AUA had statistically significant lower odds of testing Ag-positive in 2019 vs. 2018, despite having the lowest reported coverage in the 2018 MDA [14]. AUA is highly urbanised compared to the other three regions, which could contribute to lower levels of transmission due to social or environmental factors, but this link remains to be explored. See Willis et al. (2020) [14] for a detailed analysis of MDA participant coverage and demographics. The low baseline for Ag prevalence in the AUA PSUs in 2018 also means that a larger relative difference is achieved for a similar absolute reduction in infection, i.e., when very few cases are observed, a small number of additional cases will have a large effect on the percentage increase/decrease in prevalence.

In 2019, Ag prevalence remained lower in participants aged 5–9-years compared to participants aged ≥10 years. This is consistent with existing evidence of higher Ag prevalence in older age groups in the Pacific region [15,21]. Higher Ag and Mf prevalence in adults supports the notion that prevalence measured in school-based TASs may not be an appropriate estimate to extrapolate to the general population, as 6–7-year-old children are less likely to be representative of the overall population.

Locating and treating infected people within a community is an important complement to MDA interventions and can help reduce the risk of clinical complications as well as ongoing transmission. Targeted sampling to efficiently find and treat the foci of residual infection has been suggested as a complementary intervention to wide-scale MDA [22,23]. Targeted strategies that rely on reactive case-finding (also referred to as snowball sampling) assume there is a high chance that infected people will be found near other infected people, i.e., that infections are clustered. The results presented here support previous findings in this area [24], and suggest that targeted sampling of household members and potentially near neighbours would be an efficient strategy for locating LF infections in people. Further investigation is needed to better understand the potential efficiency gains from targeted sampling for on-the-ground LF surveillance. Similarly, the findings from this study, that MDA may have been less effective in reducing Ag prevalence in males than in females, particularly in the older age group, suggest that post-MDA follow-up surveillance should target this demographic for locating and treating residual infection.

Results from a concurrent survey using the molecular xenomonitoring (MX) of mosquitoes demonstrated that, in Samoa, the 2018 MDA decreased pathogen infection prevalence in mosquitoes in the short term [16]. Given the short time between the human surveys in 2018 and 2019 (approximately 5–6 months), it is possible that there was a reduction in active LF infections in humans, with insufficient time for the corresponding drop in Ag prevalence to be detected. However, there is evidence that MDA drugs have an impact on killing or sterilising adult worms as well as Mf [25], and gaps remain in our knowledge of the antigen profile in relation to the infection cycle. A survey 7-months post-MDA therefore still provides useful evidence on the impact of MDA interventions on LF infection. Nonetheless, the delay in Ag response highlights a potential limitation of Ag as an LF surveillance tool, and supports the use of complementary approaches such as Mf or MX surveillance, which may provide more sensitive indicators of ongoing transmission [16,26]. However, the practicalities and costs of each method also need to be considered, particularly in settings where there is limited access to clinical microbiologists, pathologists, and entomologists.

The timing of the surveys will also have underestimated any change in Mf prevalence from 2018 to 2019, as the first survey took place shortly after the triple-drug MDA. While this would not be expected to have any impact on Ag levels, Mf is cleared quickly following treatment [27] and the 2018 Mf prevalence presented is not an accurate baseline (Supplementary Table S1). A comparison of Mf prevalence between the two years has therefore not been included here. A further limitation on the interpretation of these results is that the 80% reported coverage from the MDA relies on self-reported data and may be subject to recall or reporting bias [14]. However, the short time frame (1–3 months) between the MDA and the 2018 survey would likely be sufficient for most participants to provide an accurate recollection of whether they took part. Additionally, the 80% reported coverage is well above the WHO-recommended coverage of ≥65% [4].

Our results show that a single round of triple-drug MDA was insufficient to break the transmission of the LF parasite. Although evidence published elsewhere attests to the efficacy of a single dose of the triple-drug regime in clearing Mf at the individual level [7,27], further MDA rounds are needed to reduce infection levels sufficiently to break transmission at a population level. Higher Ag and Mf prevalence in those aged ≥10 years, and the presence of substantial clustering of Ag at the household level, suggests that targeted surveillance strategies for these groups are needed to support LF elimination programs.

## Figures and Tables

**Table 1:** Sample size and demographic characteristics of the study population in the 2018 survey (1–3 months post-triple-drug MDA) [15] and 2019 survey 6–8 months post-triple-drug MDA). Results are presented for all 35 primary sampling units (PSUs), and separately for randomly and purposively selected PSUs as well as combined.

	All PSUs (*n* = 35)		Randomly Selected PSUs (*n* = 30)		Purposively Selected PSUs (*n* = 5)	
	2018	2019	2018	2019	2018	2019
**Participants** Total participants	3940	4290	3413	3654	527	636
Participants per PSU Mean (range)	112.6 (75–128)	122.6 (98–153)	113.8 (99–128)	121.8 (98–149)	105.4 (75–117)	127.2 (104–153)
**Households** Total households sampled	499	547	437	472	62	75
Households per PSU Mean (range)	14.3 (6–20)	15.6 (12–19)	14.9 (9–20)	15.7 (12–19)	13.3 (6–15)	15.0 (14–16)
Household size Mean (range)	4.8 (1–26)	4.8 (1–20)	4.8 (1–26)	4.7 (1–20)	5.2 (1–13)	5.6 (1–17)
**Age**						
**Participants aged 5–9 years** Mean (range)	55.5 (36–75)	60.7 (37–76)	56.2 (40–75)	60.8 (45–76)	51.2 (36–59)	60.4 (37–74)
Convenience survey	44.1 (29–61)	47.0 (17–60)	44.5 (29–61)	48.1 (28–59)	41.2 (30–49)	43.8 (17–60)
Household survey	11.4 (5–22)	13.3 (4–24)	11.7 (5–22)	12.7 (4–24)	10.0 (6–13)	16.6 (13–23)
**Participants aged ≥10 years** Mean (range)						
Household survey	57.1 (39–73)	61.9 (48–81)	57.6 (43–73)	61.0 (48–81)	54.2 (39–59)	66.8 (59–80)
**Sex**						
Male participants (%)	1927 (48.9)	2058 (48.0)	1682 (49.3)	1746 (47.8)	245 (46.5)	312 (49.1)
Female participants (%)	2013 (51.1)	2232 (52.0)	1731 (50.7)	1908 (52.2)	282 (53.5)	324 (50.9)
**Region**						
AUA participants (%)	668 (17.0)	667 (15.5)	668 (19.6)	667 (18.3)	-	-
NWU participants (%)	1628 (41.3)	1724 (40.2)	1286 (37.7)	1322 (36.2)	342 (64.9)	402 (63.2)
ROU participants (%)	885 (22.5)	1026 (23.9)	810 (23.7)	922 (25.2)	75 (14.2)	104 (16.4)
SAV participants (%)	759 (19.3)	873 (20.3)	649 (19.0)	743 (20.3)	110 (20.9)	130 (20.4)

## Data Availability

Data used in this paper were collected during field surveys in Samoa. Communities in Samoa are small (some with less than 200 inhabitants) and, so, sharing individual level data could enable identification of individual participants, therefore violating the conditions of the study’s ethics approval. For requests relating to data access, please contact the Human Ethics Department at the University of Queensland (humanethics@research.uq.edu.au), citing protocol 2021/HE000895. All relevant data at the primary sampling unit level has been included in the Supplementary Materials.

## Supplementary Materials

The following supporting information can be downloaded at: https://www.mdpi.com/article/10.3390/tropicalmed9120311/s1, Table S1. Ag and Mf prevalence in 30 randomly selected primary samples units (PSUs) in Samoa in 2018 (1.5–3.5 months post triple-drug MDA) and 2019 (6–8 months post triple-drug MDA). Table S2. Ag and Mf prevalence in 35 primary sampling units (PSUs) in 2019 in Samoa. Standardised by age and gender and adjusted for survey design. Table S3. Ag and Mf prevalence in 5 purposively selected primary samples units (PSUs) in Samoa in 2018 (1.5–3.5 months post triple-drug MDA) and 2019 (6–8 months post triple-drug MDA). Table S4. Clustering of Ag-positive participants in Samoa in 2018 and 2019 at the regional, PSU and household level.
