# Supplementary material for: Epidemiology of Lymphatic Filariasis Antigen and Microfilaria in Samoa, 2019: 7–9 Months Post Triple-Drug Mass Administration
Source: Trop Med Infect Dis. 2024 Dec 23;9(12):311. doi: 10.3390/tropicalmed9120311 (PMC11680324; doi:10.3390/tropicalmed9120311)
Supplement: Supplementary file 1 [file tropicalmed-09-00311-s001.zip › TMID SAM19_MAYFIELD Supplementary_V2.pdf]

Supplementary Material

Epidemiology of Lymphatic Filariasis Antigen and Microfilaria in Samoa, 2019: 7-9 Months Post Triple-Drug Mass Administration

Helen J. Mayfield[1], Harriet Lawford[1], Benn Sartorius[1], Patricia M. Graves[2], Sarah Sheridan[3], Therese Kearns[4], Shannon M. Hedtke[5], Katherine Gass[6], Take Naseri[7], Robert Thomsen[7], Colleen L. Lau[1].

[1] Centre for Clinical Research, The University of Queensland, Brisbane, QLD 4006, Australia
[2] College of Public Health, Medical and Veterinary Sciences, James Cook University, Cairns 4878, Australia
[3] School of Public Health and Community Medicine, University of New South Wales,[4] Menzies School of Health Research, Charles Darwin University,
[5] Department of Environment and Genetics, La Trobe University, Bundoora, VIC 3086, Australia
[6] Neglected Tropical Diseases Support Centre, The Task Force for Global Heath, Decatur, Georgia, United States of America
[7] Ministry of Health, Apia, Samoa

Supplementary S1 Table. Ag and Mf prevalence in 30 randomly selected primary samples units (PSUs) in Samoa in 2018 (1-3 months post triple-drug MDA) and 2019 (7-9 months post triple-drug MDA).

Supplementary S2 Table. Ag and Mf prevalence in 35 primary sampling units (PSUs) in 2019 in Samoa. Standardised by age and gender and adjusted for survey design.

Supplementary S3 Table. Ag and Mf prevalence in 5 purposively selected primary samples units (PSUs) in Samoa in 2018 (1-3 months post triple-drug MDA) and 2019 (7-9 months post triple-drug MDA).

Supplementary S4 Table. Clustering of Ag-positive participants in Samoa in 2018 and 2019 at the regional, PSU and household level

Supplementary S1 Table. Ag and Mf prevalence in 30 randomly selected primary samples units (PSUs) in Samoa in 2018 (1-3 months post triple-drug MDA) and 2019 (7-9 months post triple-drug MDA). Table reports results overall, as well as by age (5-9 years, ≥10-years), sex (male, female) and region (Apia Urban Area-AUA, Northwest Upolu-NWU, Rest of Upolu-ROU, Savai'i-SAV). Includes adjusted odds ratios of testing positive to antigen in 2019 compared to 2018 (reference value) Change in Mf prevalence is not reported due to the timing of surveys.

		**Antigen (Ag)**			**Microfilaria (Mf)**	
		**Ag prevalence**		**Odds Ratio**	**Mf prevalence**	
		**2018**	**2019**	**2019-2018**	**2018**	**2019**
	**Total participants sampled**	**3333**	**3626**		**3413**	**3654**
**All participants**	number testing positive, n (%)	91 (2.7)	86 (2.4)		13 (0.4)	17 (0.5)
	Adjusted prevalence (%, 95% CI)	3.9 (2.7-5.6)	4.1 (2.7-5.9)	0.84 (0.50-1.42)	0.8 (0.3-1.8)	0.8 (0.3-1.6)
**Age**						
**Participants aged 5-9 years**	number testing positive, n (%)	24 (1.4)	16 (0.9)		1 (0.1)	1 (0.6)
	Adjusted prevalence (%, 95% CI)	1.2 (0.7-2.0)	1.1 (0.5-2.2)	0.90 (0.39-2.08)	0.0 (0.0-0.3)	0.1 (0.0-1.0)
**Participants aged ≥10 years**	number testing positive, n (%)	67 (4.0)	70 (3.9)		12 (0.7)	16 (0.9)
	Adjusted prevalence (%, 95% CI)	4.7 (3.0-7.0)	4.1 (2.6-6.2)	0.83 (0.47-1.47)	1.1 (0.5-2.4)	0.9 (0.4-1.9)
**Sex**						
**Male participants**	number testing positive, n (%)	54 (3.3)	64 (3.7)		7 (0.4)	13 (0.8)
	Adjusted prevalence (%, 95% CI)	4.7 (3.3-6.6)	6.7 (4.4-9.8)	1.20 (0.75-1.92)	0.9 (0.4-1.8)	1.3 (0.5-2.8)
**Female participants**	number testing positive, n (%)	37 (2.2)	22 (1.2)		6 (0.4)	4 (0.2)
	Adjusted prevalence (%, 95% CI)	3.1 (1.6-5.4)	1.3 (0.5-2.7)	0.40 (0.16-1.02)	0.7 (0.1-2.3)	0.3 (0.1-0.8)
**Region**						
**Apia Urban Area (AUA)**	number testing positive, n (%)	17 (2.6)	5 (0.8)		-	1 (0.2)
	Adjusted prevalence (%, 95% CI)	4.4 (1.96-8.38)	1.5 (0.37-4.07)	**0.26[3] (0.08-0.79)**	-	0.1 (0.0-0.55)
**Northwest Upolu (NWU)**	number testing positive, n (%)	72 (4.5)	77 (4.5)		15 (0.9)	21 (1.2)
	Adjusted prevalence (%, 95% CI)	4.8 (3.01-7.12)	4.9 (2.45-8.52)	0.75 (0.35-1.58)	1.5 (0.72-2.78)	1.0 (0.31-2.48)
**Rest of Upolu (ROU)**	number testing positive, n (%)	15 (1.7)	18 (1.8)		2 (0.2)	2 (0.2)
	Adjusted prevalence (%, 95% CI)	1.6 (0.62-3.36)	3.9 (1.83-7.26)	2.00 (0.69-5.81)	0.4 (0.10-1.04)	0.4 (0.04-1.48)
**Savai'i (SAV)**	number testing positive, n (%)	18 (2.5)	39 (4.5)		1 (0.1)	8 (0.9)
	Adjusted prevalence (%, 95% CI)	2.7 (0.74-6.83)	5.8 (2.12-12.18)	**1.85[2] (1.33-2.58)**	0.2 (0.01-1.16)	1.5 (0.26-4.82)

[1] Change from 2018 – 2019 Significant at <0.001

[2] Change from 2018 – 2019 Significant at <0.01

[3] Change from 2018 – 2019 Significant at <0.05

Supplementary S2 Table. Ag and Mf prevalence in 35 primary sampling units (PSUs) in 2019 in Samoa. Standardised by age and gender and adjusted for survey design.

PSU number	Village	Antigen (Ag) prevalence % (95% CIs)	Microfilaria (Mf) Prevalence % (95% CIs)
**Apia Urban Area (AUA)**			
1	Vaivase Tai	None observed	None observed
2	Vaiala Tai + Vaiala Uta	None observed	None observed
3	Avele + Letava	0.7 (0.0-4.6)	0.7 (0.0-4.6)
4	Vaimea + Fugalei	5.6 (1.9-12.3)	None observed
5	Vaimoso	4.4 (1.6-9.4)	None observed
**Northwest Upolu (NWU)**			
6	Vaitoloa	None observed	None observed
7	Letogo	None observed	None observed
8	Vaiusu	9.4 (5.0-15.8)	3.1 (0.9-7.8)
9	Puipaa	1.6 (0.1-6.5)	None observed
10	Ulululoa	2.0 (0.3-6.7)	2.0 (0.3-6.7)
11	Vaitele Fou	3.4 (1.0-8.3)	None observed
12	Lotosoa	13.1 (7.7-20.3)	3.9 (1.2-8.9)
13	Nuu	1.3 (0.1-5.2)	None observed
14	Tuanai	1.4 (0.1-5.5)	None observed
15	Fasitoo Uta	8.6 (4.2-15.0)	2.3 (0.4-6.7)
16	Vailuu Tai	4.1 (1.2-9.8)	None observed
17	Leauvaa	0.8 (0.0-4.3)	0.3 (0.0-3.5)
18	Fasitoo Tai*	13.6 (8.3-20.6)	3.6 (1.1-8.3)
19	Faleasiu*	12.3 (7.6-18.6)	4.5 (1.8-9.1)
**Rest of Upolu (ROU)**			
20	Laulii*	13.9 (8.2-21.5)	4.9 (1.7-10.5)
21	Fusi	5.2 (2.0-10.7)	0.7 (0.0-4.2)
22	Faleseela	2.6 (0.5-7.3)	2.6 (0.5-7.3)
23	Manono Uta	2.5 (0.6-6.5)	None observed
24	Salani + Utulaelae	2.9 (0.7-7.6)	None observed
25	Mutiatele + Saleaaumua	None observed	None observed
26	Falefa	8.8 (4.6-15.0)	None observed
27	Musumusu + Faleapuna	None observed	None observed
28	Salua*	6.2 (2.3-12.8)	None observed
**Savai'i (SAV)**			
29	Lalomalava + Safua	1.9 (0.3-6.3)	None observed
30	Lano	None observed 0.0	None observed
31	Safotu	0.3 (0.0-3.5)	None observed
32	Sataua	2.8 (0.7-7.6)	None observed
33	Sagone	7.7 (3.8-13.8)	2.4 (0.5-6.7)
34	Papa+Tafua	18.6 (12.4-26.3)	4.9 (1.9-10.2)
35	Salelolonga*	12.7 (7.5-19.8)	2.7 (0.6-7.2)

Supplementary S3 Table. Ag and Mf prevalence in 5 purposively selected primary samples units (PSUs) in Samoa in 2018 (1-3 months post triple-drug MDA) and 2019 (7-9 months post triple-drug MDA). Table reports results overall, as well as by age (5-9 years, ≥10-years), sex (male, female) and region (Apia Urban Area-AUA, Northwest Upolu-NWU, Rest of Upolu-ROU, Savai'i-SAV). Includes adjusted odds ratios of testing positive to antigen in 2019 compared to 2018 (reference value) Change in Mf prevalence is not reported due to the timing of surveys.

		**Antigen (Ag)**			**Microfilaria (Mf)**	
		**Ag prevalence**		**Odds Ratio**	**Mf prevalence**	
		**2018**	**2019**	**2019-2018**	**2018**	**2019**
**All participants**	**Total participants sampled**	**527**	**636**		**527**	**636**
	number testing positive, n (%)	31 (6.0)	53 (8.4)		5 (1.0)	15 (2.4)
	Adjusted prevalence (%, 95% CI)	10.0 (7.2-13.4)	14.9 (13.7-16.0)	1.41 (0.85-2.35)	1.6 (0.8-2.9)	4.3 (3.4-5.4)
**Age**						
**Participants aged 5-9 years**	number testing positive, n (%)	4 (1.6)	9 (3.0)		-	1 (0.3)
	Adjusted prevalence (%, 95% CI)	2.1 (0.8-4.2)	4.2 (1.3-9.8)	2.19 (0.77-6.20)	-	0.5 (0.1-1.9)
**Participants aged ≥10 years**	number testing positive, n (%)	27 (10.2)	44 (13.3)		5 (1.9)	14 (4.2)
	Adjusted prevalence (%, 95% CI)	11.4 (7.7-16.1)	14.3 (11.4-17.6)	1.33 (0.78-2.31)	1.8 (0.8-3.7)	4.7 (2.8-7.2)
**Sex**						
**Male participants**	number testing positive, n (%)	17 (7.0)	28 (9.0)		2 (0.8)	7 (2.3)
	Adjusted prevalence (%, 95% CI)	11.7 (10.3-13.3)	**19.4**[1] **(16.9-22.1)**	1.48 (1.13-1.93)	1.0 (0.1-3.6)	4.0 (2.5-6.0)
**Female participants**	number testing positive, n (%)	14 (5.1)	25 (7.8)		3 (1.1)	8 (2.5)
	Adjusted prevalence (%, 95% CI)	8.2 (4.0-14.6)	10.0 (7.3-13.3)	1.36 (0.58-3.15)	2.3 (1.4-3.6)	4.7 (2.3-8.6)

[1] Change from 2018 – 2019 Significant at <0.001

Supplementary S4 Table. Intracluster correlation coefficient (ICC) values of Ag-positive participants in Samoa in 2018 and 2019 at the regional, PSU and household level. A higher ICC indicates greater clustering.

	All PSUs		Randomly selected		Purposively selected	
	**2018**	**2019**	**2018**	**2019**	**2018**	**2019**
**Regional ICC (95% CI)**	0.01 (0.00 -0.56)	0.02 (0.00-0.72)	0.00 (0.00-1.00)	0.00 (0.00-1.00)	0.00 (0.00-1.00)	0.02 (0.00-0.96)
**PSU ICC (95% CI)**	0.17 (0.07-0.37)	0.23 (0.12-0.41)	0.17 (0.06-0.40)	0.20 (0.08-0.40)	0.01 (0.00-1.00)	0.02 (0.00-0.96)
**Household ICC (95% CI)**	0.45 (0.31-0.61)	0.51 (0.37-0.64)	0.46 (0.30-0.63)	0.43 (0.28-0.60)	0.31 (0.10-0.66)	0.41 (0.20-0.66)
